# Transactions of the Medical Association of the State of Alabama

**Published:** 1855-08

**Authors:** 


ART. VIII—*Transactions of the Medical Association of the State of Alabama*, at its Eighth Annual Session, begun and held in the city of Mobile, February, 5, 6, and 7, 1855.

---

These transactions both in size and quality of contents give evidence that there are a large number of industrious men in the ranks of the profession in Alabama.

The volume afiords something besides the President's Address, the Annual Oration, and the Valedictory—the routine bill of fare at most Associations—it contains a number of reports and cases, and some among them are of much practical interest. The Reports on Indiginous Botany bring into notice a number of new medicinal plants, which we hope will be systematically experimented with by the members of the Association, and the results made known at some future meeting.

Dr. F. E. Gordon, read a paper on the treatment of Dysentery, from which we make the following extract for the benefit of those of our readers who are daily encountering that oftentimes unmanagable affection, premising that we ourselves have adopted the epsom salts plan with much success in a large number of cases.

There are some points connected with the treatment of dysentery that deserve special attention. In the first place, an attack of the most violent character, in a plethoric subject, can be strangled at once by free *serous* evacuations, amounting to hypercatharsis, followed immediately by morphine enemata. Some care and judgment are required in the selection of proper cases for such *active* treatment. Milder cases are treated successfully by saline purgatives sufficient to produce fœculent operations, succeeded by anodynes—or, in many instances, with the following mixture:

℞. Sulph. magnes., ℨj; tinct. opii., ℨj; aqua font., ℥iv.—M. Dose a table-spoonful every three hours.

Cases that have run on for ten days or more and resisted ordinary treatment, where the discharges have assumed a pasty grumous aspect may be relieved by injections of nitrate of silver, ten grs. to the oz. of water. In administering this remedy it is best, I think, to use but the one oz. of fluid and to take precaution to prevent its absorption. Although the pain is, usually, very great upon its first introduction into the bowel, tolerance is sometimes established and it would be retained, unless washed out by a large mucilaginous enema. This should always be done if the silver is retained beyond ten minutes. The enema of nitrate of silver should not be repeated oftener than once in twenty-four hours, though it does not prevent the repetition of anodyne and mucilaginous injections in the intervals between its administration. Another important point is "to feed your patient." It is truly surprising how well the stomach does its duty when a continuous structure is so gravely affected. Dr. Graves insists ably upon feeding the fevers of Ireland, but fortunately, having no maculated fever here, we have escaped the task. In no disease, however, of this climate, is feeding so imperatively demanded as in dysentery. Egg-nog seems well calculated to supply the albumen drained away in this disease.

Collapses occasionally occur suddenly in dysentery, and what is strange, in most of the cases that I have observed, when the bloody discharges were giving way to more natural evacuations. They are generally fatal—more so I think, than in most other diseases. I would judge that in about seven hundred cases of this disease, treated in this vicinity in the last four years, collapse has occurred five times—only one of these has recovered. Small doses of calomel and laudanum, one gr. of the former to five drops of the latter, were administered every fifteen minutes, as recommended by Dr. Ayre in cholera asphyxia, in addition to the stimulants usually given. The calomel was withdrawn after fifteen grs. had been taken, but the opium was largely increased, and had to be continued for several days. What agency the mercury had in effecting the recovery I leave for others to say.

Dr. Nott, of Mobile, who does the principal part of the surgery of South Alabama and East Mississippi, who had but recently joined the Association, made few a remarks on surgery, which, being based on a large experience extending over many years are entitled to attention. He says:

The atmosphere around Mobile Bay, (I presume the same remark would apply to the gulf coast of the United States generally) is peculiarly favorable to the success of surgical operations. Compound and complicated fractures which are considered by best authorities as cases for amputation, generally do well here—amputations are much more successful than in any printed statistics, and so with all other surgical cases.

In amputations of the extremities, I almost invariably adopt the flap operation, and have every reason to be satisfied with it, except in amputations below the knee. In operations below the knee, the old circular operation is unquestionably the best; when one large heavy flap is made from the calf, the weight is so great that firm adhesions form with difficulty and cures are tedious.

I beg leave to call the attention of the profession to a pathological fact connected with *cataracts* of Alabama, which I think worthy of more full investigation. We are informed by all writers on diseases of the eye, that the cataracts of adults, and particularly those of middle and advanced age, are always hard, and for this reason, the operation of extraction is generally preferred to that of depression or absorption.

I have now been practicing in Mobile nearly twenty years, and during this time, have had occasion to operate very frequently for cataract—very few of these have been congenital, and very few under adult age, and though my statistics have not been carefully kept, I am satisfied that four out of five of the cases operated on, have been soft, and not a few milky. To this fact, I attribute a degree of success in my operations, which is remarkable, for I do not pretend to any merits as an oculist. I have always commenced my operations upon the presumption that the cataract was to be a hard one, and if so, my plan is to displace it in the usual way. I generally find the lens too soft to bear the pressure of the needle, and have broken it up and left it to be absorbed *in situ*. I really approach a case of cataract with more confidence of success than any capital operation in surgery. The question arises, is there anything in our climate or other local circumstances which can explain this peculiarity of our cataracts, or is it chance which has thrown so large a portion in my way. I should be glad to have the experience of others on this point.

*Calculus* is a rare disease in South Alabama; I have operated on but one case of stone in the last twelve months. Since my residence in Mobile, I have operated on but twelve cases and heard of but one other presented for operation during that time. The operation selected has been uniformly the lateral, and with the gorget—all have been successful. One died from pleurisy, from imprudent exposure, a month after the operation, while fairly convalescent. It is worthy of remark, that the calculi operated on in Mobile, give an average of size, far beyond what I have seen recorded elsewhere. One operated on by myself, was as large as an orange, and another by my friend Dr. Levert, was not much less; they both had to be crushed before they could be extracted, several others required also to be crushed. Out of the small number extracted by myself in Mobile, four will average higher than any four in the immense collection of Dr. Dudley; I examined his collection with care several years ago and was surprised at the fact.

The apparatus which I have used for twenty-five years, for fracture of thigh, is a modification of the double inclined plane, and this apparatus is now generally adopted in our city; if the management of this apparatus is properly understood it is simple in application, more comfortable to the patient than any other, and more certain to prevent deformity.

I present to the Association an instrument for excision of tonsils.

which simplifies the operation very much; it consists of a pair of strong scissors with a short curve and two little hooks on the blades to catch the parts excised. The operation may be compared to snuffing a candle, and is quite as easy. This instrument is now manufactured by George Tieman, No. 63, Chatham street, New York.

The report of Dr. Troy, on the Diseases of Cahaba and its Vicinity, is well written, and abounds in interesting facts. Touching the origin of malarial fevers he says:

I have endeavored by the closest observation to detect the connection if there is any, which exists between the seasons—heat, rain, winds, etc., and the generation of malaria, using the term to signify the cause which produced our fall fevers. But so far, the result has been purely negative. One cannot predict that any given combination of atmospheric causes will or will not be followed by fevers.

The last summer has furnished some remarkable instances of the fallacy of the generally received opinions on the production of malaria.

Of all the causes the action of intense heat of the sun, upon a soil saturated with moisture and full of organic matter, is considered the most efficient. From the 1st to the 20th of June this year, it rained heavily almost every day; the earth was saturated, and ponds and water courses filled to overflowing. On the 21st it became clear and hot, and so remained for ten or twelve days, no such heat had been experienced here for many years. The thermometer stood above 95° every day, and was seldom much below 90° even in the coolest part of the night. Evaporation went on with almost incredible rapidity; ponds, creeks and stagnant water alike disappeaed, and left their bottoms exposed to the broiling sun. But no bad results followed. There was no marked increase of sickness, nearly every severe case we saw during this time could be clearly traced to the direct heat of the sun, and there were comparatively few, even of these cases. There would no doubt have been many cases of sun stroke if it had not been for the prudence of the planters, who as a general rule, did not allow their hands to go in the sun, between the hours of 11 A. M. and 4 P. M. But these precautions would have been fruitless to preserve them from the action of malaria, which whatever is its nature, exerts its most pernicious influence, early in the morning and late in the evening, when the negroes were most exposed.

Of our white population, very few were sick at all. It was not till the weather had considerably moderated, and again become showery, that fevers became common.

The first two weeks of July, were hot, but not so oppressive as the two which preceded them, and there were no doubt more important changes in the atmosphere than that of temperature. There was complete revolution in the direction of the wind, and probably in the whole electrical condition of the air. During the hot dry days of June, the wind was steady from the north, and felt hot and suffocating. It was always hotter than the still air in the shade; a thermometer carried into a fresh draught of air would rise several degrees. Now it was from the south, cooler than the still air and more refreshing. There were frequent light showers and the weather was comparatively pleasant. Immediately after this change, cases of fever began to be more numerous, and they continued to increase steadily and rapidly as long as it continued, which was about two weeks. The second week of July was, therefore, the sickliest one of the whole year; it then became again very dry and hot, and so continued with no variation which seemed to effect the health of the people till fall. It was never very sickly after the middle of July. The severest cases of the season occurred in latter part of October. The first killing frost was on the 14th of November, but malarious fevers had in a great measure disappeared before it came, and our town remained almost perfectly free from endemic diseases to the end of the year, with the exception of one family who had typhoid fever.

The treatment of malarious fever is vastly more simple and effectual than it was a few years ago, and the plan which I prefer is still more simple than any recommended in the books. Early in the season it was seldom necessary to give any evacuating medicine. During the fever if there was much pain or restlessness, it was my usual practice to give a full dose of Dover's powders with two or three grs. of calomel, which was repeated in three or four hours if the first did not relieve. More than two doses were seldom required. I preferred this to any other diaphoretic, and have never seen it disagree with a patient or do harm. Care was then taken that the patient should be fully under the influence of quinine at the time the next paroxysm was expected. This was accomplished by giving fifteen or twenty grains in two, three or four doses according to the time there was to spare. If there was much fever, or no remission, quinine was always combined with opium or one of its preparations, and generally with a full dose of camphor also. It was very seldom that there would be another paroxysm. The small quantity of calomel combined with the Dover's powder usually acted upon the bowels freely enough the next day, and when it did not, no cathartic was given unless there was some urgent necessity for it. The action of a cathartic, about the time of an expected chill, always renders its prevention less sure. Calomel in these small doses acts merely by stimulating the hepatic secretion, and can hardly be considered a cathartic. I

had but little experience with emetics, in the treatment of bilious fever this year, but I may safely say that I have never administered one in this disease that I have not regretted it, and was never called after one had been administered, that I did not think the case would have done better without it. This is no doubt a much more sweeping condemnation than they deserve, but it is my experience. I still promote vomiting when the indications for it are very strong, but always with like result; the patient is no better for the puking, the more quiet the stomach can be kept, the better they do with me. Of another practice which has recently been recommended I can speak favorably. It is to give a full dose of quinine, say grs. x or xij combined with ℨss, each of the tinctures of opium and camphor, *in the chill.* I adopted this plan in about a dozen cases this season, and in every one with good result. In several the paroxysm was cut short; the patient being in a perspiration within an hour, in others the paroxysm lasted from three to six hours, but never was attended by pain or restlessness. In no case was there any return of the chill or fever, though in one or two no other medicine was given.

Later in the fall more active treatment was necessary. Mercury was absolutely necessary in most of the cases, grs. ij or iij of calomel, combined with an equal quantity of camphor, and repeated every three or four hours, till x or xij grs. of each were taken, followed by quinine in smaller and more frequent doses than in the summer. In these cases there was little or no remission, and it was necessary to keep up the effect of the medicine constantly for twenty-four hours, so as to be sure to include the time of the rise of fever. In these cases, we always gave quinine in solution, using generally equal parts of paregoric and spts. nitre as a menstruum. The cases which occurred during the excessively hot weather of June were attended by almost apoplectic symptons. They recovered more readily under the use of stimulants than any other treatment. Neither emetics, cathartics nor direct depletion would afford any relief; on the contrary, I believe the symptoms were always aggravated by them. Quinine, carb. ammonia and camphor given in combination, or alternated, would very quickly relieve patients who seemed almost beyond the hope of medicine.

*Coup de Soleil* having formed the subject of an article on another page we will make the following extract from Dr. Troy's report, in which it will be observed he pursues the plan of treatment we have recommended in this disease:

I saw three slight cases of sun stroke; the patients becoming suddenly blind and falling to the ground. In only one, was consciousness completely lost, and in that one only for a few minutes. They all quickly recovered under the use of very active stimulants as brandy, camphor, opium and capsicum. This treatment of coup de soliel is the principal one here, and is almost uniformly successful where death does not take place before any treatment can be instituted, and it contrasts very favorably with the depleting practice which regards as congestive apoplexy, a disease which is more probably merely a state of nervous exhaustion. But the treatment was adopted by the planters because they found it successful, and not from any theoretical notions of the nature of the malady they were treating.

The Annual Oration is not so free from errors as it should be, but among so many productions that are creditable and scientific, it is not worth while to single out the unimportant defects of what should always be the least important portion of the transactions of any Medical Society, namely, its addresses. D. W. Y.

